# N-benzyladriamycin-14-valerate (AD 198) exhibits potent anti-tumor activity on TRAF3-deficient mouse B lymphoma and human multiple myeloma

**DOI:** 10.1186/1471-2407-13-481

**Published:** 2013-10-16

**Authors:** Shanique K E Edwards, Carissa R Moore, Yan Liu, Sukhdeep Grewal, Lori R Covey, Ping Xie

**Affiliations:** 1 Department of Cell Biology and Neuroscience, Rutgers University, 604 Allison Road, Nelson Labs Room B336, Piscataway, NJ 08854, USA; 2 Graduate Program in Molecular Biosciences, Rutgers University, Piscataway, NJ 08854, USA; 3 Rutgers Cancer Institute of New Jersey, New Brunswick, USA

**Keywords:** AD 198, Non-Hodgkin lymphoma, Multiple myeloma, TRAF3, c-Myc

## Abstract

**Background:**

TRAF3, a new tumor suppressor identified in human non-Hodgkin lymphoma (NHL) and multiple myeloma (MM), induces PKCδ nuclear translocation in B cells. The present study aimed to evaluate the therapeutic potential of two PKCδ activators, N-Benzyladriamycin-14-valerate (AD 198) and ingenol-3-angelate (PEP005), on NHL and MM.

**Methods:**

*In vitro* anti-tumor activities of AD 198 and PEP005 were determined using TRAF3^-/-^ mouse B lymphoma and human patient-derived MM cell lines as model systems. *In vivo* therapeutic effects of AD 198 were assessed using NOD SCID mice transplanted with TRAF3^-/-^ mouse B lymphoma cells. Biochemical studies were performed to investigate signaling mechanisms induced by AD 198 or PEP005, including subcellular translocation of PKCδ.

**Results:**

We found that AD 198 exhibited potent *in vitro* and *in vivo* anti-tumor activity on TRAF3^-/-^ tumor B cells, while PEP005 displayed contradictory anti- or pro-tumor activities on different cell lines. Detailed mechanistic investigation revealed that AD 198 did not affect PKCδ nuclear translocation, but strikingly suppressed c-Myc expression and inhibited the phosphorylation of ERK, p38 and JNK in TRAF3^-/-^ tumor B cells. In contrast, PEP005 activated multiple signaling pathways in these cells, including PKCδ, PKCα, PKCϵ, NF-κB1, ERK, JNK, and Akt. Additionally, AD198 also potently inhibited the proliferation/survival and suppressed c-Myc expression in TRAF3-sufficient mouse and human B lymphoma cell lines. Furthermore, we found that reconstitution of c-Myc expression conferred partial resistance to the anti-proliferative/apoptosis-inducing effects of AD198 in human MM cells.

**Conclusions:**

AD 198 and PEP005 have differential effects on malignant B cells through distinct biochemical mechanisms. Our findings uncovered a novel, PKCδ-independent mechanism of the anti-tumor effects of AD 198, and suggest that AD 198 has therapeutic potential for the treatment of NHL and MM involving TRAF3 inactivation or c-Myc up-regulation.

## Background

In the United States, lymphoid neoplasms are the 5th most common human cancer with over 70,000 new cases annually, resulting in approximately 21,000 deaths per year [1-3]. For unknown reasons, the annual incidence of non-Hodgkin lymphoma (NHL) has doubled since the 1970s [3]. Mature B cell neoplasms account for over 90% of lymphoid tumors worldwide. Despite recent advances in treatment, many types of human B cell lymphomas remain incurable, highlighting a clear need for new preventative and therapeutic strategies [1-3]. Identification and validation of novel genetic risk factors and critical oncogenic pathways are imperative for further translational efforts. In keeping with these goals, recent studies from our laboratory and others have identified TRAF3, a critical determinant of B cell survival [4,5], as a novel tumor suppressor in a variety of human B cell lineage neoplasms. Homozygous deletions and inactivating mutations of the *Traf3* gene have been identified in NHL, including splenic marginal zone lymphoma (MZL), B cell chronic lymphocytic leukemia (B-CLL) and mantle cell lymphoma (MCL), as well as multiple myeloma (MM) and Waldenström’s macroglobulinemia (WM) [6-9].

TRAF3, a member of the TRAF family of cytoplasmic adaptor proteins, has E3 ubiquitin ligase activity [10,11]. It was first identified as an interacting protein shared by CD40 (a receptor pivotal for B cell activation) and LMP1 (an Epstein-Barr virus-encoded oncogenic protein) [12]. TRAF3 also binds to receptors for the critical B cell survival factor BAFF, including BAFF-R, TACI and BCMA. Initial studies of mice homozygous for a null allele of *Traf3* showed that they died by day 10 after birth with severe progressive runting and massive loss of splenic cellularity [13]. To circumvent limitations imposed by this early mortality and, more specifically, to explore the functions of TRAF3 in B lymphocytes, we recently generated mice bearing a conditional allele of TRAF3 [4]. By characterizing mice that have the *Traf3* gene specifically deleted in B lymphocytes (B-TRAF3^-/-^ mice), we found that TRAF3 deletion causes vastly prolonged survival of mature B cells independent of BAFF, which eventually leads to B lymphoma development in mice [4,14]. Resting splenic B cells from these mice show increased levels of active NF-κB2 but decreased levels of nuclear PKCδ [4,5]. Using B lymphoma cells derived from B-TRAF3^-/-^ mice as model systems, we demonstrated that oridonin, a pharmacological inhibitor of NF-κB, and lentiviral vectors of NF-κB2 shRNAs induce apoptosis in cultured TRAF3^-/-^ B lymphoma cells [14]. These studies identified constitutive NF-κB2 activation as one oncogenic pathway in TRAF3^-/-^ B cells.

Interestingly, available evidence suggests that the second signaling pathway downstream of TRAF3 inactivation, the reduced PKCδ nuclear translocation, may also contribute to prolonged B cell survival. First, the splenic B cell compartment of PKCδ^-/-^ mice is greatly expanded [15,16], similar to that observed in B-TRAF3^-/-^ mice [4,5] and BAFF or NF-κB2 transgenic mice [17,18]. Second, the physiological B cell survival factor, BAFF, also reduces PKCδ nuclear levels in splenic B cells [19]. In light of these observations, the present study sought to evaluate the therapeutic potential of PKCδ activation in TRAF3^-/-^ tumor B cells using two pharmacological activators of PKCδ, N-Benzyladriamycin-14-valerate (AD 198) and ingenol-3-angelate (PEP005) [20-25]. We found that AD 198 exhibited potent *in vitro* and *in vivo* anti-tumor activity on TRAF3^-/-^ tumor B cells, while PEP005 displayed contradictory anti- or pro-tumor activities on different cell lines. Our detailed mechanistic investigation revealed that AD 198 and PEP005 acted through distinct biochemical mechanisms. Interestingly, although PKCδ was identified as the principal target of AD 198 in other cancer cells, AD 198-induced apoptosis of tumor B cells was mediated through PKCδ-independent suppression of c-Myc expression. In contrast, PEP005 activated multiple signaling pathways in these cells, including PKCδ, PKCα, PKCϵ, NF-κB1, ERK, JNK, and Akt. Furthermore, we extended the investigation of AD 198 to TRAF3-sufficient malignant B cells, and found that AD198 also potently inhibited the proliferation/survival and suppressed c-Myc expression in TRAF3-sufficient mouse and human B lymphoma cell lines. Taken together, our findings suggest that AD 198 has therapeutic potential for the treatment of NHL and MM involving TRAF3 inactivation or Myc up-regulation.

## Methods

### Mice

TRAF3^flox/flox^CD19^+/Cre^ (B-TRAF3^-/-^) and TRAF3^flox/flox^ (littermate control, LMC) mice were generated as previously described [4]. NOD SCID mice (Jackson Laboratory, stock number: 001303, strain name: NOD.CB17-Prkdc^scid^/J) were used as recipients in B lymphoma transplantation and *in vivo* drug treatment experiments. All mice were kept in specific pathogen-free conditions in the Animal Facility at Rutgers University, and were used in accordance with NIH guidelines and under an animal protocol (Protocol # 08–048) approved by the Animal Care and Use Committee of Rutgers University.

### Cell lines and cell culture

Human MM cell lines 8226 (contains bi-allelic TRAF3 deletions), KMS11 (contains bi-allelic TRAF3 deletions) and LP1 (contains inactivating TRAF3 frameshift mutations) were generously provided by Dr. Leif Bergsagel (Mayo Clinic, Scottsdale, AZ). Human B lymphoma cell lines Daudi (Burkitt’s lymphoma), Ramos (Burkitt’s lymphoma), and JeKo-1 (mantle cell lymphoma) were obtained from American Type Culture Collection (ATCC, Manassas, VA). All human MM and B lymphoma cell lines were cultured as previously described [7]. Mouse B lymphoma cell lines A20.2J and CH12.LX were generously provided by Dr. Gail Bishop (University of Iowa, Iowa City, IA), and m12.4.1 was purchased from ATCC. All mouse B lymphoma cell lines were cultured as we described [14]. Generation of TRAF3^-/-^ mouse B lymphoma cell line 27–9.5.3 was described previously [14].

Mouse B lymphoma cell line 105–8.1B6 was generated from ascites harvested from a B-TRAF3^-/-^ mouse (mouse ID: 105–8) [14]. Briefly, ascitic cells (5 × 10^5^ cells/well) were plated in 24-well plates in mouse B cell media [4] containing 10% fetal bovine serum. After being cultured for 2 months, 4 actively proliferating clones were expanded, passaged, and frozen. The 105–8.1B6 clone had been cultured for 5 months without obvious changes in morphology or growth rate, and was used for drug treatment experiments.

Mouse B lymphoma cell line 115–6.1.2 was derived from splenic B lymphoma of another B-TRAF3^-/-^ mouse (mouse ID: 115–6) [14]. Briefly, Primary splenic B lymphoma cells harvested from mouse 115–6 were serially passaged in NOD SCID mice twice. B lymphoma cells (5 × 10^5^ cells/well) harvested from transplanted NOD SCID mice were plated in 24-well plates in mouse B cell media containing 10% fetal bovine serum. After being cultured for 1 month, 8 actively proliferating clones were expanded, passaged, and frozen. The 115–6.1.2 clone had been cultured for 5 months without obvious changes in morphology or growth rate, and was used for drug treatment experiments.

### Antibodies and reagents

Polyclonal rabbit Abs against RelB, NF-κB1, RelA, c-Rel, HDAC1, and PKCδ were purchased from Santa Cruz Biotechnology (Santa Cruz, CA). Polyclonal rabbit Abs specific for NF-κB2, c-Myc, phosphorylated PKCδ, PKCα, PKCϵ, caspase 3, and COX IV, and Abs against total or phosphorylated ERK, p38, JNK, and Akt, were from Cell Signaling Technology (Beverly, MA). Anti-actin Ab was from Chemicon (Temecula, CA). HRP-labeled secondary Abs were purchased from Jackson ImmunoResearch Laboratories, Inc. (West Grove, PA). Tissue culture supplements including stock solutions of sodium pyruvate, L-glutamine, and non-essential amino acids and Hepes (pH 7.55) were from Invitrogen (Carlsbad, CA). Oridonin was purchased from CalBiochem (EMD Chemicals, Gibbstown, NJ). AD 198, PEP005, 3-(4,5-dimethylthiazol-2-yl)-2,5-diphenyltetrazolium bromide (MTT), propidium iodide (PI), hexadimethrine bromide (polybrene), and rabbit anti-FLAG Abs were purchased from Sigma-Aldrich Corp. (St. Louis, MO). Allophycocyanin (APC)-conjugated-anti**-**Thy1.1 Ab was obtained from eBioscience (San Diego, CA). TRIzol reagent was from Invitrogen, and the High Capacity cDNA Reverse Transcription Kit was purchased from Applied Biosystems (Carlsbad, CA). DNA oligonucleotide primers were obtained from Integrated DNA Technologies (Coralville, IA). Pfu UltraII was purchased from Agilent (Santa Clara, CA).

### MTT assay

For primary TRAF3^-/-^ B lymphomas, cells were cultured for 4 days to obtain cleaner tumor cell populations for MTT assays. Tumor cells (1 × 10^5^ cells/well) were plated in 96-well plates in the absence or presence of AD 198 or PEP005 of various concentrations. Twenty-four hours later, total viable cell numbers were measured using the MTT assay as described [14,26]. Possible influences caused by direct MTT-drug interactions were excluded by studies in a cell-free system. Wells with untreated cells or with medium alone were used as positive and negative controls, respectively. Total viable cell number curves were plotted as a percentage of untreated control cells [14,24,27].

### Measurement of apoptosis

Cell apoptosis was assessed by both cell cycle analyses of the sub-G1 population and caspase 3 cleavage assays. For cell cycle analyses, cells (3 × 10^5^ cells/well) were cultured in 24-well plates in the absence or presence of appropriate doses of AD 198 or PEP005 for 24 hours, and fixed with ice-cold 70% ethanol. Cell cycle distribution was determined by propidium iodide (PI) staining followed by flow cytometry as previously described [4,28]. For caspase 3 cleavage assays, total cellular proteins were prepared from cells at different time points after treatment with AD 198, and cleavage of caspase 3 was subsequently examined by immunoblot analysis.

### Lymphoma transplantation and drug treatment of NOD SCID mice

TRAF3^-/-^ mouse B lymphoma cell line 27–9.5.3 cells (3 × 10^6^ cells per mouse) were *i.p.* injected into NOD SCID mice. On day 2 post transplantation, mice were divided into 3 cohorts for administration with drugs or with vehicle. Mice were *i.p.* injected with 150 μl (for a 20 g mouse) of AD 198 (5 mg/kg), oridonin (7.5 mg/kg), or vehicle (90% PBS and 10% DMSO). Drug or vehicle injections were carried out three times a week for 2 weeks. Transplanted NOD SCID mice were monitored daily for tumor development, or signs of illness or discomfort, including weight loss, enlarged lymph nodes or abdomen, labored breathing, hunched posture, and paralysis [29]. Histopathological examination of diseased mice was performed as previously described [14,29].

### Total protein lysates

Cells (for mouse B lymphoma, 10 × 10^6^ cells per condition; for human MM, 3.5 × 10^6^ cells per condition) were treated with appropriate doses of AD 198 or PEP005 for different time periods. Cell pellets were lysed in 200 μl of 2X SDS sample buffer (0.0625 M Tris, pH6.8, 1% SDS, 15% glycerol, 2% β-mercaptoethanol and 0.005% bromophenol blue), sonicated for 30 pulses, and boiled for 10 minutes.

### Cytosolic and nuclear extracts

Cells (for mouse B lymphoma, 10 × 10^6^ per condition; for human MM, 5 × 10^6^ per condition) were treated with appropriate doses of AD 198 or PEP005 for different time periods. Cytosolic and nuclear extracts were prepared from the cells as described [4,14]. Briefly, cells were washed with ice-cold PBS, swelled in 500 μl of hypotonic Buffer A (10 mM HEPES, pH7.5, 10 mM KCl, 0.1 mM EDTA and 0.1 mM EGTA with protease and phosphatase inhibitors) for 15 minutes, and then lysed by addition of 31.5 μl of 10% NP-40. Lysates were centrifuged at 13,000 rpm for 5 minutes, and the supernatants were harvested as cytosolic extracts. The pellets were incubated with 100 μl of hypertonic Buffer C (20 mM HEPES, pH7.5, 0.4 M NaCl, 1 mM EDTA and 1 mM EGTA with protease and phosphatase inhibitors), vigorously agitated at 4°C for 45 minutes, and centrifuged at 13,000 rpm for 10 minutes at 4°C. The resulting supernatants in Buffer C were harvested as nuclear extracts. One-fifth volume of 5× SDS sample buffer was added into each cytosolic or nuclear extracts, which were subsequently boiled for 10 minutes.

### Fractionation of cytosol, nuclei and membranes

Cells (for mouse B lymphoma, 30 × 10^6^ cells per condition; for human MM, 15 × 10^6^ cells per condition) were treated with appropriate doses of AD 198 or PEP005 for 5, 10 or 30 minutes. Cytosol, nuclei and membranes were fractionated from cells as previously described [23,24]. Briefly, cells were washed with ice-cold PBS, swelled in 700 μl of hypotonic Buffer M (10 mM HEPES, pH7.5, 10 mM KCl, 1 mM EDTA, 0.1 mM EGTA and 250 mM sucrose with protease and phosphatase inhibitors) on ice for 10 minutes, and then homogenized in a Dounce homogenizer. Cell lysis was checked by trypan blue uptake. Nuclei were isolated by centrifugation at 2,000 rpm for 10 minutes at 4°C. The supernatants were transferred to new tubes, and centrifugation at 13,000 rpm for 45 minutes was used to separate cytosol (supernatant) from membrane (pellet) fractions. One-fifth volume of 5× SDS sample buffer was added into the cytosol fraction. The pellets of nuclei and membranes were lysed in 200 μl of 2× SDS sample buffer respectively, and sonicated for 10 pulses. All protein samples were subsequently boiled for 10 minutes.

### Immunoblot analysis

Aliquots of total protein lysates, cytosolic and nuclear extracts, or fractions of cytosol, nuclei and membranes were separated by SDS-PAGE, and electroblotted onto nitrocellulose membranes (ProTran, Schleicher & Schuell BioScience, Keene, NH). Immunoblot analysis was performed using various antibodies as previously described [30]. Images of immunoblots were acquired using a low-light imaging system (LAS-4000 mini, FUJIFILM Medical Systems USA, Inc., Stamford, CT).

### Taqman assay of c-Myc mRNA expression

Cells (for mouse B lymphoma, 10 × 10^6^ cells per condition; for human MM, 5 × 10^6^ cells per condition) were treated with appropriate doses of AD 198 for different time periods. Total cellular RNA was extracted using TRIzol reagent (Invitrogen) according to the manufacturer’s protocol. Complementary DNA (cDNA) was prepared from RNA using High Capacity cDNA Reverse Transcription Kit (Applied Biosystems). Quantitative real-time PCR was performed using TaqMan Gene Assay kit (Applied Biosystems). TaqMan primers and probes (FAM-labeled) specific for mouse or human *c-Myc* were used in the PCR reaction to detect *c-Myc* mRNA in mouse B lymphoma and human MM cells, respectively. Each reaction also included primers and the probe (VIC-labeled) specific for mouse or human β-actin mRNA, which served as endogenous control. Relative mRNA expression levels of c-Myc were analyzed using the Sequence Detection Software (Applied Biosystems) and the comparative Ct (ΔΔCt) method following the manufacturer’s procedures. For each biological sample, duplicate PCR reactions were performed.

### Generation of lentiviral c-Myc expression vectors

The c-Myc coding cDNA sequence was cloned from the human MM cell line 8226 cells by reverse transcription and high-fidelity PCR using primers human c-Myc-F (5′- ACG ATG CCC CTC AAC GTT AG -3′) and human c-Myc-R (5′- TCC TTA CGC ACA AGA GTT CC -3′). The high-fidelity polymerase Pfu UltraII (Agilent) was used in the PCR reaction. The c-Myc coding cDNA sequence was subcloned into the lentiviral expression vector pUB-eGFP-Thy1.1 [31] (kindly provided by Dr. Zhibin Chen, the University of Miami, Miami, FL) by replacing the eGFP coding sequence with the c-Myc coding sequence. To facilitate the differentiation of transduced c-Myc from endogenous c-Myc, we engineered an N-terminal FLAG tag in frame with the c-Myc coding sequence, and generated a lentiviral expression vector of FLAG-tagged c-Myc (pUB-FLAG-c-Myc-Thy1.1). The human c-Myc coding sequence and the lentiviral expression vectors were verified by DNA sequencing.

### Lentiviral packaging and transduction of human MM cells

Lentiviruses of the pUB-FLAG-c-Myc-Thy1.1 vector or an empty lentirival vector pUB-Thy1.1 were packaged and titered as previously described [31,32]. The pUB lentiviral expression vectors have an expression cassette of the marker Thy1.1, and thus allow the transduced cells to be analyzed by Thy1.1 immunofluorescence staining followed by flow cytometry. Human MM cell lines 8226 and LP1 cells were transduced with the packaged lentiviruses at a multiplicity of infection (MOI) of 1:5 (cell:virus) in the presence of 8 μg/ml polybrene [31]. On day 4 post transduction, the transduction efficiency of cells was analyzed by flow cytometry. Transduced cells were subsequently analyzed for c-Myc protein expression and responses to treatment with AD 198.

### Statistics

Statistical analyses were performed using the Prism software (GraphPad, La Jolla, CA). Survival curves were generated using the Kaplan-Meier method, and were compared using a log-rank (Mantel-Cox) test to determine whether differences are significant. For other experiments, statistical significance was assessed by Student *t* test. *P* values less than 0.05 are considered significant.

## Results

### Differential effects of AD 198 and PEP005 on TRAF3^-/-^ tumor B cells

**Figure 1:**
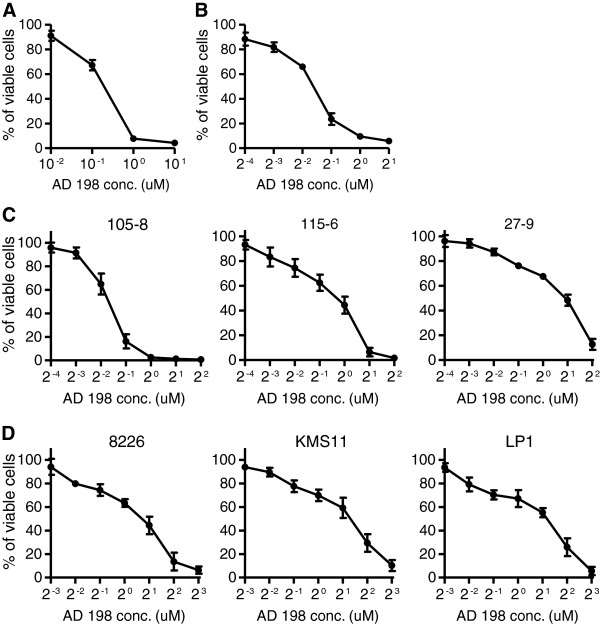
**AD 198 exhibited potent anti-proliferative/survival-inhibitory effects on TRAF3**^**-/- **^**mouse B lymphoma and human MM cells.** Tumor B cells were treated with various concentrations of AD 198 for 24 h. Total viable cell numbers were subsequently determined by MTT assay. **(A and B)** Effects on primary splenic B lymphoma cells harvested from diseased B-TRAF3^-/-^ mice. Panel **A** shows the activity of AD 198 examined with a wide range of doses (1:10 serial dilutions). Panel **B** shows refined dose-dependent effects of AD 198 (examined at 1:2 serial dilutions). Similar results were also obtained with primary B lymphoma cells purified from ascites, cervical and mesenteric LNs of several individual B-TRAF3^-/-^ mice with spontaneous tumors. **(C)** Effects on TRAF3^-/-^ B lymphoma cell lines derived from 3 individual B-TRAF3^-/-^ mice, including 105–8.1B6 (105–8), 115–6.1.2 (115–6), and 27–9.5.3 (27–9). **(D)**  Effects on human patient-derived MM cell lines with TRAF3 bi-allelic deletions or frameshift mutations, including 8226, KMS11 and LP1. The graphs depict the results of three independent experiments with duplicate samples in each experiment (mean ± SEM).

We recently reported that decreased PKCδ nuclear translocation is a feature of both premalignant TRAF3^-/-^ B cells and primary TRAF3^-/-^ B lymphomas derived from B-TRAF3^-/-^ mice [4,14]. It has also been shown that PKCδ^-/-^ mice have greatly increased numbers of B cells and that inhibition of PKCδ nuclear translocation contributes to BAFF-mediated survival of peripheral B cells [15,16,19]. These previous studies suggest that PKCδ nuclear translocation is a downstream signaling event of TRAF3 and induces apoptosis in B cells. This prompted us to test the possibility that restoration of PKCδ nuclear translocation may have therapeutic potential in TRAF3^-/-^ tumor B cells. We thus evaluated the effects of two pharmacological activators of PKCδ, AD 198 and PEP005, on primary TRAF3^-/-^ B lymphomas harvested from diseased B-TRAF3^-/-^ mice. Our results of MTT assays demonstrated that AD 198 exhibited potent anti-proliferative/survival-inhibitory effects on primary TRAF3^-/-^ B lymphoma cells in a dose-dependent manner (effective doses: 0.1 to 1 μM) (Figure 1A and 1B). In sharp contrast, PEP005 stimulated the proliferation of primary TRAF3^-/-^ B lymphoma cells at the dose range (10 to 100 nM) that has been previously shown to display anti-tumor activity on myeloid leukemia cells [23,24] (Additional file 1: Figure S1A and 1B).

We then compared the effects of AD 198 and PEP005 on three TRAF3^-/-^ B lymphoma cell lines derived from different individual B-TRAF3^-/-^ mice, including 105–8.1B6, 115–6.1.2 and 27–9.5.3 [14]. These 3 cell lines differ in their Ig VDJ sequences, malignant states, and metastatic capabilities. The 105–8.1B6 cell line is IgM+, does not contain somatic hypermutation (SHM) in the Ig VDJ region, and develops peritoneal and splenic B lymphomas within 4 months when transplanted into NOD SCID recipient mice. The 115–6.1.2 cell line is IgM+, contains SHM in the Ig VDJ region, and develops peritoneal and splenic B lymphomas within 2 months when transplanted into NOD SCID recipient mice, which occasionally metastasize to the kidney and liver. The 27–9.5.3 cell line is IgG+, contains SHM in the Ig VDJ region, and develops peritoneal and splenic B lymphomas, which often metastasize to the kidney, liver and lung within 3 weeks when transplanted into NOD SCID recipient mice. We found that AD 198 consistently exhibited potent anti-proliferative/survival-inhibitory effects on all 3 TRAF3^-/-^ B lymphoma cell lines in a dose-dependent manner (effective doses: 0.25 to 4 μM) (Figure 1C). In contrast, PEP005 displayed contradictory effects on these 3 cell lines. PEP005 induced the proliferation of 105–8.1B6 cells, killed 115–6.1.2 cells, and did not affect 27–9.5.3 cells at the dose range of 12.5 to 100 nM (Additional file 1: Figure S1C).

To extend the clinical relevance of our findings, we further examined the effects of AD 198 and PEP005 on three human patient-derived MM cell lines with TRAF3 deletions or mutations: 8226, KMS11 and LP1. Both 8226 and KMS11 cell lines contain bi-allelic deletions of the *Traf3* gene, while LP1 cell line has frameshift mutations of *Traf3*[14]. These cell lines represent naturally occurring TRAF3^-/-^ tumor B cells. Our results of MTT assays showed that the responses of the 3 human MM cell lines to AD 198 and PEP005 recapitulated those of mouse TRAF3^-/-^ B lymphoma cell lines (Figure 1D and Additional file 1: Figure S1D). Together, these data indicate that AD 198 exhibits potent anti-proliferative/survival-inhibitory effects, whereas PEP005 displays divergent effects on TRAF3^-/-^ mouse B lymphoma cells and human MM cells.

### AD 198 but not PEP005 induced apoptosis in TRAF3^-/-^ tumor B cells

**Figure 2:**
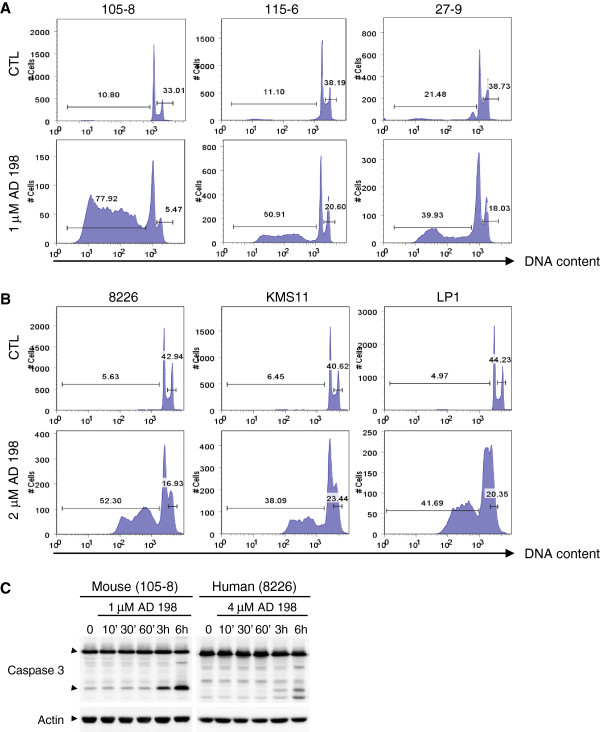
**AD 198 induced apoptosis in TRAF3**^**-/- **^**tumor B cells. (A and B)** Cell cycle distribution determined by PI staining and flow cytometry. TRAF3^-/-^ mouse B lymphoma cell lines **(A)** or human MM cell lines **(B)** were cultured in the absence or presence of AD 198 of indicated concentration for 24 h, and then fixed. Fixed cells were stained with PI and analyzed by FACS. Representative histograms of PI staining are shown, and percentage of apoptotic cells (DNA content < 2n; sub-G1) and proliferating cells (2n < DNA content ≤ 4n; S/G2/M) are indicated. Results are representative of 3 independent experiments. **(C)** Western blot analysis of caspase 3 cleavage. Cells were cultured in the absence or presence of AD 198 of indicated concentration. Total cellular lysates were prepared at indicated time points, and then immunoblotted for caspase 3, followed by actin. Immunoblots of actin were used as loading control. Results are representative of three independent experiments. Similar results were also obtained with other cell lines examined.

To understand the mechanism of AD 198 and PEP005, we first performed cell cycle analysis using PI staining followed by flow cytometry. We found that AD 198 induced TRAF3^-/-^ mouse B lymphoma cells and human MM cells to undergo apoptosis, as demonstrated by the drastic increase of the sub-G1 population with DNA content < 2n (Figure 2A and 2B). AD 198 also inhibited the proliferation of TRAF3^-/-^ tumor B cells, as shown by the marked decrease of the population at the S/G2/M phase (2n < DNA content ≤ 4n) (Figure 2A and 2B). In contrast, PEP005 increased the population at the S/G2/M phase in mouse 105–8.1B6 and human 8226 cells, but induced the apoptotic population and decreased the population at the S/G2/M phase in mouse 115–6.1.2 cells. PEP005 did not have significant effects on the cell cycle distribution in mouse 27–9.5.3 as well as human KMS11 and LP1 cell lines (Additional file 1: Figure S2A and 2B). We next determined whether AD 198 induced the activation of the key effector caspase, caspase 3, involved in apoptosis. We found that AD 198 induced the rapid activation of caspase 3, as evidenced by the cleavage of caspase 3 as early as three hours after treatment with AD 198 in TRAF3^-/-^ mouse B lymphoma and human MM cell lines (Figure 2C). Collectively, our data demonstrate that AD 198 but not PEP005 induces rapid apoptosis in TRAF3^-/-^ tumor B cells.

### AD 198 exhibited potent *in vivo* anti-tumor activity on TRAF3^-/-^ mouse B lymphomas

The potent *in vitro* anti-proliferative/apoptosis-inducing effects of AD 198 led us to further assess its *in vivo* therapeutic potential. We recently reported that B-TRAF3^-/-^ mice display a long and varied latency (9 ~ 18 months) in developing B lymphomas [14]. Therefore, B-TRAF3^-/-^ mice are not ideal for drug treatment experiments. In this study, we used NOD SCID mice transplanted with the highly malignant TRAF3^-/-^ B lymphoma cell line 27–9.5.3 as model systems for *in vivo* drug treatment experiments. We also included the study of oridonin, an inhibitor of NF-κB2 and NF-κB1 activation, which also exhibits robust *in vitro* tumoricidal activity on primary TRAF3^-/-^ B lymphomas harvested from diseased B-TRAF3^-/-^ mice [14].

**Figure 3:**
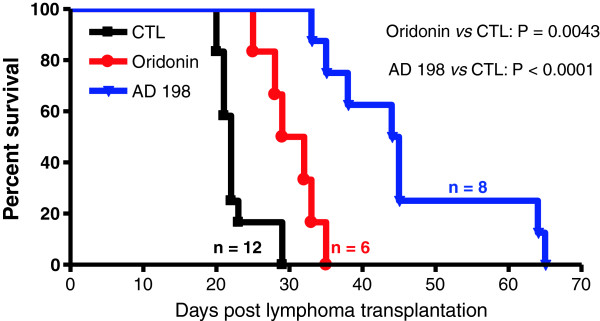
**AD 198 and oridonin exhibited potent anti-tumor activity on transplanted TRAF3**^**-/- **^**B lymphomas in NOD SCID mice.** TRAF3^-/-^ mouse B lymphoma cell line 27–9.5.3 cells (3 x 10^6^ per mouse) were *i.p.* injected into NOD SCID recipient mice. On day 2 post transplantation, mice were divided into 3 cohorts for administration with drugs or with vehicle. Mice were *i.p.* injected with 150 μl (for a 20 g mouse) of AD 198 (5 mg/kg, n = 8), oridonin (7.5 mg/kg, n = 6), or vehicle (90% PBS and 10% DMSO; CTL, n = 12). Drug or vehicle injections were carried out three times a week for 2 weeks. Transplanted NOD SCID mice were monitored daily for tumor development as described in the Methods. Survival curves of mice were generated using the Kaplan-Meier method. *P* value of AD 198 *vs.* CTL is <0.0001, and *P* value of oridonin *vs.* CTL is 0.0043, as determined by the Mantel-Cox log-rank test.

In the absence of drug treatment, transplantation of 27–9.5.3 cells (3 × 10^6^ cells/mouse) caused rapid B lymphoma development in NOD SCID mice, which killed the mice at 23 ± 3 (Mean ± SD) days post-transplantation (Figure 3). Necropsy revealed that B lymphomas were not only developed in the peritoneal cavity and spleen, but also often metastasized to the kidney, liver and lung. Treatment of transplanted NOD SCID mice with oridonin significantly prolonged the survival of mice to 30 ± 3.7 days post-transplantation (p = 0.0043, determined by the Mantel-Cox log-rank test). However, metastasis to the kidney, liver and lung was also common in oridonin-treated mice. Interestingly, administration of AD 198 into transplanted NOD SCID mice vastly extended the survival of mice to 46 ± 12 days post-transplantation (p < 0.0001, determined by the Mantel-Cox log-rank test). Furthermore, B lymphomas were typically localized in the peritoneal cavity, and metastasis to the kidney, liver and lung was rare (2 out of 8) in AD 198-treated mice. Interestingly, consistent with previous studies [33-36], we did not observe any adverse effects of AD 198 at the dose examined (5 mg/kg) in mice, such as weight loss or liver damage. Taken together, our results demonstrate that both AD 198 and oridonin exhibit *in vivo* anti-tumor activity on TRAF3^-/-^ mouse B lymphomas.

### AD 198 induced PKCδ cleavage, while PEP005 induced PKC translocation in TRAF3^-/-^ tumor B cells

**Figure 4:**
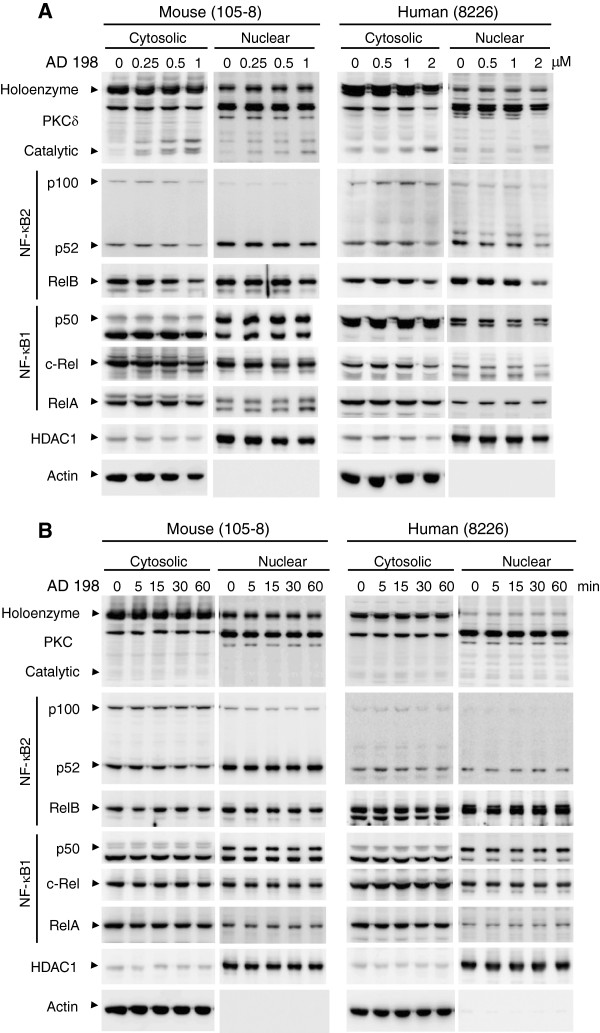
**AD 198 did not affect PKCδ nuclear translocation or NF-κB activation. (A)** Dose-dependent effects of AD 198. Mouse or human tumor B cells were cultured in the absence or presence of various concentrations of AD 198 for 6 h. **(B)** Time-dependent effects of AD 198. Mouse or human tumor B cells were cultured in the absence or presence of AD 198 (1 μM for 105–8 cells and 2 μM for 8226 cells) for indicated time periods. Cytosolic and nuclear extracts were prepared as described in the Methods. Proteins were immunoblotted for PKCδ, NF-κB2 (p100 – p52), RelB, NF-κB1 (p50), c-Rel, and RelA, followed by HDAC1 and actin. Immunoblots of actin and HDAC1 were used as loading control for cytosolic and nuclear proteins, respectively. Results are representative of three independent experiments. Similar results were also obtained with other cell lines examined.

Both AD 198 and PEP005 have been previously shown to induce the subcellular translocation of PKCδ in myeloid leukemia cells, which mediates the anti-leukemic effects of these two drugs [20,21,23,24]. PKCδ nuclear translocation also regulates B cell apoptosis [15,16,19]. We thus compared the effects of AD 198 and PEP005 on PKCδ nuclear translocation using cytosolic and nuclear extracts, which were prepared by the same method described in our previous studies [4,14]. Surprisingly, neither AD 198 nor PEP005 increased the nuclear levels of PKCδ at 6 hours after treatment in TRAF3^-/-^ tumor B cells (Figure 4A and Additional file 1: Figure S3A). Interestingly, we noticed that AD 198 but not PEP005 induced the cleavage of cytosolic PKCδ from the 78 kDa holoenzyme to the 40 kDa catalytic fragment at 6 hours after treatment in a dose dependent manner (Figure 4A and Additional file 1: Figure S3A). We next determined the time-course effects of AD 198 and PEP005 on PKCδ nuclear translocation or cleavage. We found that neither AD 198 nor PEP005 increased the nuclear levels or cleavage of PKCδ at 5 to 60 minutes after treatment (Figure 4B and Additional file 1: Figure S3B). In these experiments, we also examined the effects of AD 198 and PEP005 on the other oncogenic pathways that we recently identified, NF-κB2 and NF-κB1 activation [14]. It has been shown that AD 198 and PEP005 promote NF-κB1 activation in breast cancer and primary acute myeloid leukemia cells [22,37,38]. No obvious effects of AD 198 were observed on nuclear levels of NF-κB2 (p52/p100) or NF-κB1 subunits (p50, c-Rel and RelA), except for a moderate inhibition of RelB levels by the highest dose of AD 198 examined (Figure 4A). The only noticeable effect of PEP005 was the increase of nuclear RelA and c-Rel levels at 15 to 60 minutes after treatment in mouse 105–8 cells (Additional file 1: Figure S3B).

**Figure 5:**
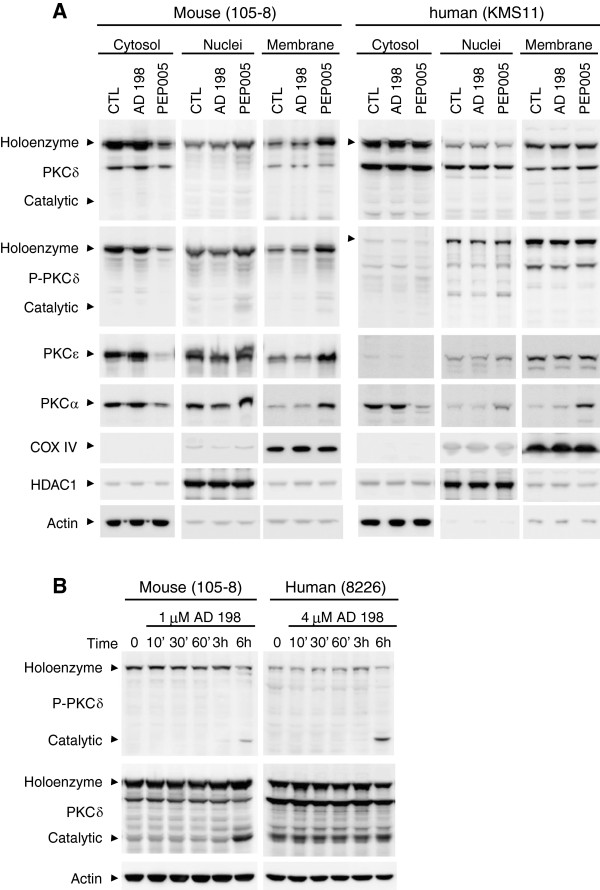
**Differential effects of AD 198 and PEP005 on PKCδ subcellular translocation and cleavage. (A)** Subcellular translocation of PKC isoforms. Mouse or human tumor B cells were cultured in the absence or presence of AD 198 or PEP005 for 10 minutes. Drug concentrations used: 1 μM AD 198 and 0.1 μM PEP005 for 105–8 cells; 4 μM AD 198 and 0.2 μM PEP005 for 8226 cells. Biochemical fractionation of cytosol, nuclei and membrane was performed as described in the Methods. Proteins were immunoblotted for PKCδ, phosphorylated-PKCδ (P-PKCδ), PKCϵ, and PKCα, followed by COXIV, HDAC1, and actin. Immunoblots of actin, HDAC1, and COX IV were used as loading control for proteins of cytosol, nuclei, or membrane (including mitochondria), respectively. Similar results were also obtained at 5 minutes or 30 minutes after treatment, and with another TRAF3^-/-^ mouse B lymphoma cell line 27–9.5.3 and another human MM cell line 8226 (except barely detectable levels of PKCα in 8226 cells). **(B)** Phosphorylation and cleavage of PKCδ. Cells were cultured in the absence or presence of AD 198 of indicated concentration. Total cellular lysates were prepared at indicated time points, and then immunoblotted for phosphorylated-PKCδ (P-PKCδ), PKCδ, followed by actin. Similar results were also obtained with other cell lines examined. Results are representative of three independent experiments.

Our unexpected results of PKCδ nuclear translocation prompted us to compare our biochemical method of nuclear extraction with the previous method used to study AD 198 and PEP005 in myeloid leukemia cells. We identified one major difference in the methods: our nuclear extracts did not include proteins localized at or associated with the nuclear membrane, but the nuclei fraction prepared in previous studies of AD 198 and PEP005 did. We thus prepared subcellular fractions of cytosol, nuclei, and membrane (including mitochondria) using the method that was previously employed in the study of AD 198 and PEP005 in myeloid leukemia cells [23,24]. As shown in Figure 5A, our results clearly demonstrated that PEP005 induced the rapid translocation of PKCδ, PKCϵ and PKCα from the cytosol to the nuclei and membranes (including mitochondria) in TRAF3^-/-^ mouse B lymphoma cells. Similarly, PEP005 induced the rapid translocation of PKCα from the cytosol to the nuclei and membranes in TRAF3^-/-^ human MM cells (Figure 5A). However, in sharp contrast, AD 198 did not affect the subcellular distribution of PKCδ, PKCϵ or PKCα in any TRAF3^-/-^ tumor B cell lines examined in this study (Figure 5A).

It is known that activation of PKCδ is not only regulated by subcellular translocation, but also modulated by phosphorylation and cleavage of PKCδ [39,40]. Subcellular translocation allows PKCδ to access its nuclear substrates (such as DNA damage response proteins DNA-dependent protein kinase, Rad9 and p53) and mitochondrial substrates (such as an anti-apoptotic protein Mcl-1 and a regulator of phospholipid movement Phospholipid Scramblase 3) [39,40]. Cleavage of PKCδ removes the N-terminal auto-inhibitory regulatory domain from the catalytic fragment of PKCδ, thereby causing activation of PKCδ in the absence of any co-factors [39,40]. Depending on the stimuli and the cellular context, phosphorylation of PKCδ may regulate its subcellular translocation, cleavage, or substrate selectivity [39,40]. In light of these previous findings, we further assessed the effects of AD 198 on the phosphorylation and cleavage of PKCδ in TRAF3^-/-^ tumor B cell lines. We found that AD 198 did not induce the phosphorylation of PKCδ from 10 minutes up to 6 hours after treatment in any TRAF3^-/-^ tumor B cell lines examined in this study (Figure 5A and 5B). Interestingly, AD 198 did induce the cleavage of PKCδ at 6 hours after treatment in TRAF3^-/-^ tumor B cells (Figures 4A and 5B). However, the induction of PKCδ cleavage occurred relatively late, and was preceded by caspase 3 activation, which was detected at 3 hours after AD 198 treatment (Figure 2C). It has been previously shown that PKCδ is a substrate of caspase 3, which cleaves the 78 kDa holoenzyme of PKCδ to generate the 40 kDa catalytic fragment of PKCδ [41]. Therefore, it is very likely that PKCδ cleavage induced by AD 198 is a consequence of caspase 3 activation in TRAF3^-/-^ tumor B cells, and is not the initiating signal that triggers the apoptosis. Taken together, our findings suggest that AD 198 induces the apoptosis of TRAF3^-/-^ tumor B cells not through the translocation or activation of its known target PKCδ, but through a distinct novel mechanism.

### Differential effects of AD 198 and PEP005 on ERK, p38 and JNK activation in TRAF3^-/-^ tumor B cells

**Figure 6:**
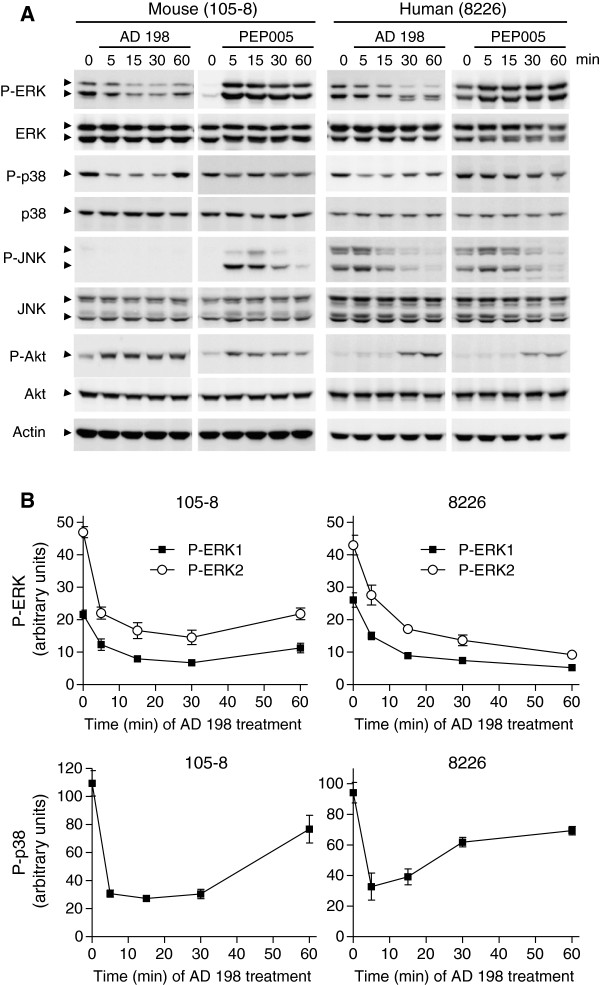
**AD 198 inhibited the phosphorylation of ERK, p38 and JNK in TRAF3**^**-/- **^**tumor B cells. (A)** Phosphorylation of MAPK and Akt analyzed by immunoblot analysis. Mouse or human tumor B cells were cultured in the absence or presence of AD 198 or PEP005 for indicated time periods. Drug concentrations used: 1 μM AD 198 and 0.1 μM PEP005 for 105–8 cells; 4 μM AD 198 and 0.2 μM PEP005 for 8226 cells. Total cellular lysates were prepared, and then immunoblotted for phosphorylated (P-) or total ERK, p38, JNK, and Akt, followed by actin. **(B)** Quantitation of ERK and p38 phosphorylation. Phosphorylated and total ERK1, ERK2, and p38 bands on immunoblots were quantitated using a low-light imaging system, and the results are presented graphically. The amount of P-ERK1, P-ERK2, or P-p38 in each lane was normalized to the intensity of the corresponding total ERK1, ERK2 or p38 band. The graphs depict ERK and p38 phosphorylation observed in three independent experiments (mean ± SD).

To gain further insights into the molecular mechanisms underlying the anti-tumor effect of AD 198 and the divergent effects of PEP005, we next sought to investigate key signaling pathways that are known to play important roles in regulating B cell survival and proliferation, including the activation of ERK, p38, JNK, and Akt. We found that AD 198 markedly and rapidly decreased the phosphorylation levels of ERK1 (p44), ERK2 (p42), and p38 in TRAF3^-/-^ mouse B lymphoma and human MM cells (Figure 6A and 6B). Inhibition of ERK and p38 phosphorylation was detected as early as 5 minutes after AD 198 treatment. AD 198 also inhibited JNK activation in TRAF3^-/-^ human MM cells. Interestingly, although Akt activation is considered as a general survival pathway, AD 198 increased the Ser473 phosphorylation and thus activation of Akt in TRAF3^-/-^ mouse B lymphoma and human MM cells. In contrast, PEP005 induced the activation of ERK, JNK and Akt in TRAF3^-/-^ mouse B lymphoma cells, and also induced ERK and Akt activation in human MM cells (Figure 6A). Taken together, our results suggest that the differential effects of AD 198 and PEP005 on tumor B cells are mediated by their distinct effects on multiple signaling pathways, including PKCδ, PKCϵ, and PKCα translocation (Figure 5A), and ERK, p38 and JNK phosphorylation (Figure 6A).

### AD 198 rapidly suppressed c-Myc expression in TRAF3^-/-^ tumor B cells

**Figure 7:**
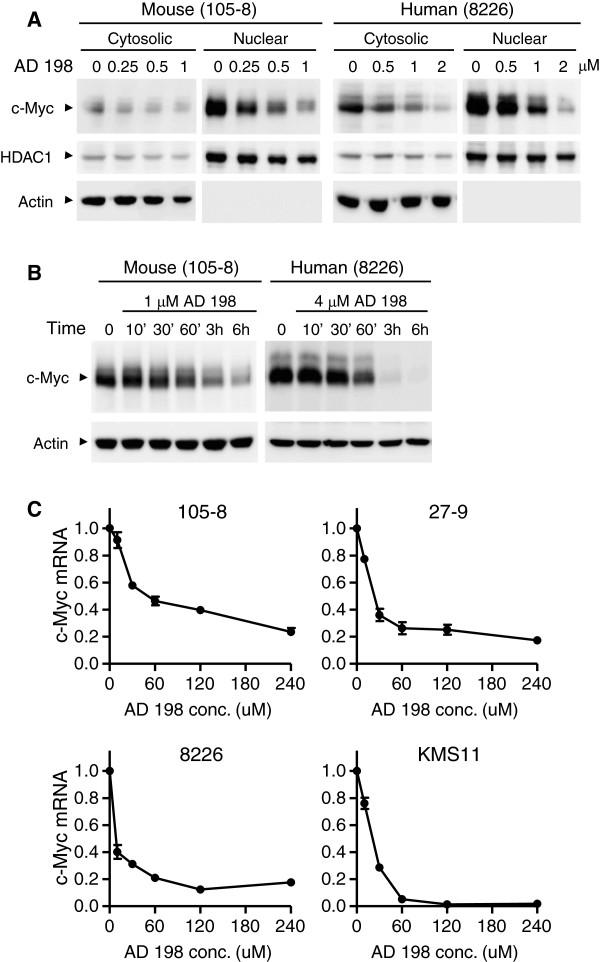
**AD 198 suppressed c-Myc expression in TRAF3**^**-/- **^**tumor B cells. (A)** Dose-dependent effects of AD 198 on c-Myc protein levels. Mouse or human tumor B cells were cultured in the absence or presence of various concentrations of AD 198 for 6 h. Cytosolic and nuclear extracts were prepared as described in the Methods. **(B)** Time-dependent effects of AD 198 on c-Myc protein levels. Mouse or human tumor B cells were cultured in the absence or presence of AD 198 (1 μM for 105–8 cells and 4 μM for 8226 cells). Total cellular lysates were prepared at indicated time points. Proteins were immunoblotted for c-Myc, followed by actin. Immunoblots of actin were used as loading control. Results of A and B are representative of three independent experiments. Similar results were also obtained with other TRAF3^-/-^ cell lines. **(C)***c-Myc* transcript levels analyzed by real time PCR. Cells were treated with AD 198 for indicated time periods. Drug concentrations used: 1 μM AD 198 for 105–8 cells; 2 μM AD 198 for 27–9 cells; 4 μM AD 198 for 8226 and KMS11 cells. Total cellular RNA was extracted and reverse transcribed. Quantitative real-time PCR was performed using TaqMan primers and probe (FAM-labeled) specific for *c-Myc*. Each PCR reaction also included primers and the probe (VIC-labeled) specific for β-actin mRNA, which served as endogenous control. Relative mRNA expression levels of *c-Myc* were analyzed using the Sequence Detection Software and the comparative Ct (ΔΔCt) method. Graphs depict the results of three independent experiments with duplicate samples in each experiment (mean ± SEM).

One known target gene of ERK, p38 and JNK signaling pathways that is especially essential for B cell survival and proliferation is c-Myc [42-44]. In light of our evidence that AD 198 inhibited ERK, p38 and JNK signaling pathways, we further investigated the effects of AD 198 on c-Myc protein levels. We found that AD 198 potently decreased protein levels of c-Myc, which was primarily localized in the nucleus, in a dose-dependent manner at 6 hours after treatment in TRAF3^-/-^ mouse B lymphoma and human MM cells (Figure 7A). We next found that AD 198 vastly inhibited c-Myc protein levels as early as 1 hour after treatment in all TRAF3^-/-^ tumor B cell lines examined in this study (Figure 7B). In contrast, PEP005 did not inhibit c-Myc protein levels in any tumor B cell lines examined (Additional file 1: Figure S3).

To understand the mechanism of AD198-mediated suppression of c-Myc protein levels, we examined the mRNA levels of c-Myc by reverse transcription and quantitative real time PCR analyses. As shown in Figure 7C, AD 198 dramatically and rapidly inhibited the mRNA levels of c-Myc in TRAF3^-/-^ mouse B lymphoma and human MM cells. Decrease in c-Myc mRNA levels was detected as early as 10 minutes after AD 198 treatment, and could completely account for the decrease in c-Myc protein levels observed in these cells. These results indicate that AD 198 potently suppresses c-Myc mRNA and protein expression in TRAF3^-/-^ tumor B cells.

### AD 198 exhibited potent anti-tumor activity and rapidly suppressed c-Myc expression in TRAF3-sufficient B lymphoma cell lines

**Figure 8:**
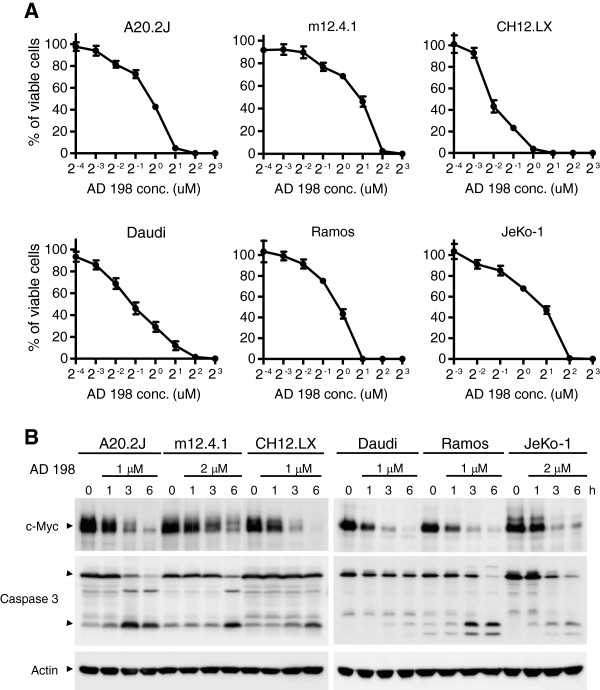
**AD 198 exhibited potent anti-tumor activity and suppressed c-Myc expression in TRAF3-sufficient mouse and human B lymphoma cell lines. (A)** Anti-tumor activity of AD 198. Mouse B lymphoma cell lines A20.2 J, m12.4.1 and CH12.LX, or human B lymphoma cell lines Daudi, Ramos and JeKo-1 were treated with various concentrations of AD 198 for 24 h. Total viable cell numbers were subsequently determined by MTT assay. The graphs depict the results of three independent experiments with duplicate samples in each experiment (mean ± SEM). **(B)** Time-dependent effects of AD 198 on c-Myc protein levels. Mouse or human B lymphoma cells were cultured in the absence or presence of AD 198 of indicated concentrations. Total cellular lysates were prepared at indicated time points. Proteins were immunoblotted for c-Myc, followed by caspase 3 and actin. Immunoblots of actin were used as loading control. Results are representative of three independent experiments.

Considering that elevated expression of c-Myc is associated with many B cell malignancies [45], we further tested the therapeutic effects of AD 198 on TRAF3-sufficient B lymphoma cell lines. These include mouse B lymphoma cell lines A20.2J, m12.4.1, and CH12.LX, and human B lymphoma cell lines Daudi (Burkitt’s lymphoma), Ramos (Burkitt’s lymphoma), and JeKo-1 (mantle cell lymphoma). Our results of MTT assays demonstrated that AD 198 also exhibited potent anti-proliferative/apoptosis-inducing effects (effective dose: 0.25 to 4 μM) on all the TRAF3-sufficient mouse and human B lymphoma cell lines examined in this study (Figure 8A). To determine whether c-Myc is also the principal target of AD 198 in TRAF3-sufficient B lymphoma cells, we examined the effects of AD 198 on c-Myc protein levels. We found that AD 198 strikingly inhibited c-Myc protein levels as early as 1 hour after treatment in all TRAF3-sufficient B lymphoma cell lines examined in this study (Figure 8B). AD 198 also induced the cleavage and activation of caspase 3 at 3 hours after treatment in these cell lines. It should be noted that c-Myc suppression precedes caspase 3 activation, suggesting that c-Myc is not the consequence, but may be the trigger of apoptosis. Together, these results indicate that AD 198 also has therapeutic potential and targets c-Myc in TRAF3-sufficient B lymphomas.

### Lentiviral vector-mediated constitutive expression of c-Myc conferred partial resistance to the anti-tumor effects of AD 198 in human MM cell lines

**Figure 9:**
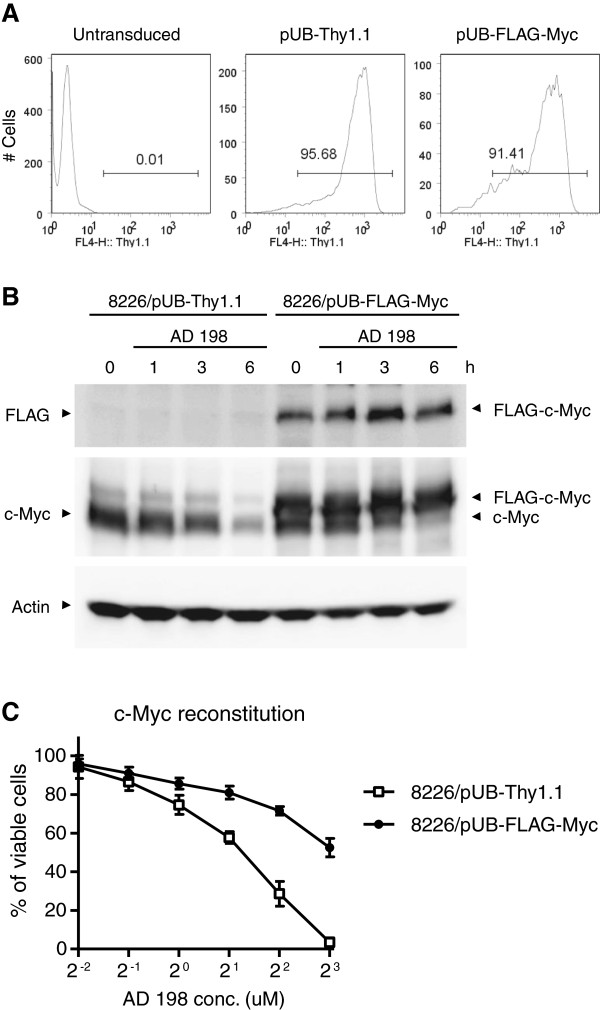
**Constitutive c-Myc expression counteracted the effects of AD 198 on the proliferation and survival of human MM cells.** The human MM cell line 8226 cells were transduced with a lentiviral expression vector of FLAG tagged c-Myc (pUB-FLAG-c-Myc-Thy1.1, labeled as pUB-FLAG-Myc in the figure). Cells transduced with an empty lentiviral expression vector (pUB-Thy1.1) were used as control in these experiments. **(A)** Transduction efficiency analyzed by Thy1.1 immunofluorescence staining and flow cytometry. Gated population (Thy1.1+) indicates cells successfully transduced with each lentiviral expression vector. Untransduced 8226 cells were used as negative control. **(B)** Time-dependent effects of AD 198 on endogenous c-Myc and transduced FLAG-c-Myc protein levels. Transduced 8226 cells (8226/pUB-Thy1.1 or 8226/pUB-FLAG-Myc) were cultured in the absence or presence of 4 μM AD 198. Total cellular lysates were prepared at indicated time points, and were immunoblotted for FLAG, c-Myc, followed by actin. Immunoblots of actin were used as loading control. Results are representative of three independent experiments. **(C)** Decreased anti-proliferative/survival-inhibitory effects of AD 198 on human MM cells with constitutive c-Myc expression. Transduced 8226 cells (8226/pUB-Thy1.1 or 8226/pUB-FLAG-Myc) were treated with various concentrations of AD 198 for 24 h. Total viable cell numbers were subsequently determined by MTT assay. The graph depicts the results of three independent experiments with duplicate samples in each experiment (mean ± SEM). Similar results were also observed in another human MM cell line LP1 cells.

To further investigate whether c-Myc suppression contributes to the anti-tumor effects of AD 198 in malignant B cells, we performed reconstitution of c-Myc expression experiments. We generated a lentiviral expression vector of FLAG-tagged human c-Myc, pUB-FLAG-c-Myc-Thy1.1, in which constitutive expression of c-Myc is driven by the ubiquitin promoter. Human MM cell lines 8226 and LP1 cells were transduced with this vector or an empty lentiviral expression vector (pUB-Thy1.1), and then analyzed for their responses to AD 198 treatment. Transduction efficiency of the lentiviral vectors was over 90% in human MM cells, as demonstrated by immunofluorescence staining and flow cytometry (Figure 9A). Following treatment with AD 198, although endogenous c-Myc protein levels were strikingly decreased, the transduced FLAG-c-Myc protein levels were not suppressed by AD 198 as shown in the immunoblots of both FLAG and c-Myc (Figure 9B). These results demonstrated that expression of the transduced FLAG-c-Myc driven by the ubiquitin promoter was not suppressed by AD 198, suggesting that AD 198 inhibits the transcription of endogenous c-Myc via its effects on the c-Myc promoter. Interestingly, we further found that constitutive expression of FLAG-c-Myc substantially counteracted the effects of AD 198 on the proliferation and survival of human MM cells (Figure 9C). Thus, our results indicate that c-Myc suppression is a major contributing factor to the anti-tumor effects of AD 198 in human MM cells.

## Discussion

We previously showed that premalignant TRAF3^-/-^ B cells and TRAF3^-/-^ B lymphomas have decreased nuclear levels of PKCδ [4,14]. This, together with the evidence that decreased nuclear translocation of PKCδ promotes B cell survival [15,16,19], prompted us to evaluate the therapeutic potential of two PKCδ activators, AD 198 and PEP005, in TRAF3^-/-^ mouse B lymphomas and human MM cells. In the present study, we report that AD 198 exhibited potent *in vitro* and *in vivo* anti-tumor activity on TRAF3^-/-^ tumor B cells, while PEP005 displayed contradictory anti- or pro-tumor activities on different cell lines. AD 198 and PEP005 induced differential effects on TRAF3^-/-^ tumor B cells through distinct biochemical mechanisms. Our detailed mechanistic study uncovered a novel PKCδ-independent mechanism of the anti-tumor effect of AD 198 that involves c-Myc suppression. Furthermore, we found that AD 198 also exhibited potent anti-tumor effects and targeted c-Myc in TRAF3-sufficient mouse and human B lymphoma cell lines. Our findings suggest that AD 198 has therapeutic potential for the treatment of NHL and MM involving TRAF3 inactivation or c-Myc up-regulation.

Ingenol-3-angelate (PEP005) is an active ingredient of the sap from *Euphorbia peplus*, which has been used for centuries in the U.K. and Australia as a traditional treatment for skin conditions, including warts, corns and skin cancers. PEP005 has now entered phase II clinical trials as a topical treatment for non-melanoma skin cancers and actinic keratoses. PEP005 is also being developed as a systemic treatment for acute myeloid leukemia in preclinical models [24,25,46]. Anti-tumor effects of PEP005 have also been demonstrated in *s.c.* inoculated melanoma, lung carcinoma, prostate cancer, cervical carcinoma, and bladder cancer [46,47]. PEP005 is structurally closely related to phorbols, and is a potent activator of novel (δ, ϵ, η, θ) and classical (α, β, γ) isoenzymes of PKC at lower concentrations (10 to 100 ng/ml) [48]. However, PKCδ is the isoform that mediates the pro-apoptotic effects of PEP005 in myeloid leukemia and colon cancer cells [23,24,49]. In these cells, PEP005 induces PKCδ translocation from the cytoplasm to the plasma membrane, nuclear membrane and mitochondrial membrane.

Interestingly, we detected PEP005-induced nuclear and membrane translocation of PKCδ, PKCα and PKCϵ in TRAF3^-/-^ tumor B cells (Figure 5A). In cancer, PKCα and PKCϵ are generally linked to proliferation or survival and thus considered as oncogenes. In contrast, PKCδ has a pro-apoptotic function in a variety of cancer cells [22,24,50]. Activation of PKC isoforms signals further downstream events, such as the activation of p38, ERK, JNK or NF-κB in melanoma, myeloid leukemia and colon cancer cells [25,27,35,50,51], which were all observed in our study of tumor B cells. In colon cancer cells, inhibition of Akt phosphorylation via a PKC-independent mechanism also contributes to the apoptotic effects of PEP005 [24,49]. In contrast, we found that PEP005 induced Akt phosphorylation in TRAF3^-/-^ tumor B cells. Therefore, the ultimate effect of cell proliferation or apoptosis induction by PEP005 depends on the balance between the levels and activities of pro-apoptotic (PKCδ) and anti-apoptotic (PKCα and PKCϵ) isoforms of PKC as well as their crosstalk with different signaling pathways (MAPKs, NF-κB, and Akt) in each tumor B cell line. Indeed, we detected varying levels of different PKC isoforms (α, δ and ϵ) in different tumor B cell lines, and this may contribute to the observed divergent responses of the cells to PEP005. Our findings thus provide new insights into the complexity of the signaling pathways controlled by PEP005 in TRAF3^-/-^ tumor B cells.

N-Benzyladriamycin-14-valerate (AD 198) is a novel semisynthetic, lipophilic anthracycline analogue with experimental anti-tumor activity superior to that of doxorubicin (DOX) [22,34,52]. Nuclear-targeted anthracycline antibiotics, such as DOX, are known to exert their anti-tumor effects primarily via DNA intercalation and topoisomerase II inhibition, thereby causing double-stranded DNA breaks and promoting apoptosis. The extranuclear-targeted AD 198, unlike DOX, only weakly binds DNA, and does not inhibit topoisomerase II [22,34,52,53]. AD 198 is structurally similar to commonly accepted ligands for the C1 regulatory domain of PKC, and binds to the diacylglycerol-binding C1b domain of classical and novel PKC isoforms [38,52,54]. The interaction between AD 198 and the C1b domain leads to the activation of PKC isoforms, especially PKCδ, and thereby promoting rapid apoptosis in a variety of cancer cells, including myeloid leukemia, breast cancer, prostate cancer, melanoma, colon cancer, and ovarian carcinoma [20,22,38,54]. PKCδ was identified as the principal target of AD 198 that mediates the apoptotic effects of AD 198 [20,22,53].

In myeloid leukemia cells, AD 198 induces the mitochondrial and membrane translocation of PKCδ, PKCα and PKCϵ [20,21,38,53]. In the present study, we found that AD 198 induced caspase 3 activation, PKCδ cleavage, and DNA fragmentation in TRAF3^-/-^ tumor B cells as previously observed in myeloid leukemia cells. However, AD 198-induced apoptosis of tumor B cells was not mediated through the activation of PKCδ, as translocation or phosphorylation of PKCδ was not detected. Additionally, translocation of PKCα or PKCϵ was not induced by AD 198 in tumor B cells either. Thus, the anti-proliferative and apoptosis-inducing effects of AD 198 on TRAF3^-/-^ tumor B cells are mediated through a novel, PKCδ-independent mechanism.

We identified c-Myc as the principal target of AD 198 in TRAF3^-/-^ and TRAF3-sufficient malignant B cells. We found that both the mRNA and protein levels of c-Myc were strikingly and rapidly suppressed by AD 198. In support of the important role of c-Myc down-regulation, we observed that lentiviral vector-mediated constitutive expression of c-Myc conferred robust resistance to the anti-proliferative/survival-inhibitory effects of AD 198 in human MM cells. The c-Myc protein is a central regulator of B cell survival and proliferation, and has a short half-life (about 20–30 minutes) [55,56]. It has been previously shown that the promoter regions of both human and mouse *c-Myc* genes contain binding sites for AP-1, a transcription factor directly activated by ERK, p38 and JNK signaling pathways [42-44]. AP-1 is also indirectly inhibited by Akt activity [42]. Interestingly, we found that AD 198 inhibited ERK, p38 and JNK activation, but promoted Akt activation in TRAF3^-/-^ tumor B cells. In this context, our results suggest that AD 198 targets c-Myc by inhibiting c-Myc transcription in tumor B cells, which is mediated through inhibition of ERK, p38 and JNK pathways as well as activation of the Akt pathway. However, we could not exclude additional mechanisms, as it has been shown that AD 198 inhibits E. coli RNA polymerase or chicken leukemic RNA polymerase activity through drug-template interaction or enzyme inactivation, respectively [57]. Regardless of the exact mechanisms, given that elevated expression of c-Myc is ubiquitously observed in many B cell malignancies [45], our findings suggest that AD 198 may have wide therapeutic application in B cell neoplasms.

It has been shown that AD 198 has anti-tumor activity superior to DOX in breast cancer, ovarian carcinoma and melanoma models [36,58], which was recapitulated in our TRAF3^-/-^ mouse B lymphomas. We previously showed that DOX did not exhibit tumoricidal activity on primary B lymphoma cells derived from B-TRAF3^-/-^ mice [14]. Here we report that AD 198 has potent anti-tumor effects on TRAF3^-/-^ mouse B lymphomas and human MM. AD 198 can also override multiple mechanisms of DOX resistance, including those mediated by p53 dysfunction, or by overexpression of the multidrug transporters (P-glycoprotein and multidrug resistance protein) or the anti-apoptotic proteins (Bcl-2, Bcl-xL and NF-κB) [20,38,53,59]. Importantly, AD 198 is also pharmacologically superior to DOX in terms of its decreased cardiotoxicity, low hematotoxicity, and the rapid rate of intracellular uptake [33-36]. The use of DOX is limited by its dose-dependent, and often irreversible cardiotoxicity. However, AD 198 does not exhibit significant cardiotoxicity or other organ toxicities at therapeutic doses, and is cardioprotective in rodent models [33-36]. In support of this notion, we demonstrated that in NOD SCID mice transplanted with TRAF3^-/-^ mouse B lymphomas, administration of AD 198 drastically extended the survival of mice and inhibited the growth and metastasis of B lymphomas. In fact, AD 198 demonstrated a higher *in vivo* potency than oridonin, an inhibitor of both NF-κB2 and NF-κB1 pathways [14]. In summary, our findings reveal a novel PKCδ-independent mechanism of AD 198 that targets c-Myc in malignant B cells, and support further clinical studies of AD 198 as an anti-cancer agent for NHL and MM.

## Conclusions

In the present study, we have uncovered a novel, PKCδ-independent mechanism of the anti-tumor effects of AD 198 that strikingly targets c-Myc in TRAF3^-/-^ tumor B cells. AD 198-induced signaling events appear to occur in the following order: AD 198 treatment → diminished phosphorylation of ERK1/2 and p38 but increased Akt phosphorylation (5 minutes) → down-regulation of c-Myc transcription (10–30 minutes) → decreased protein levels of c-Myc (1 hour) → caspase 3 activation (3 hours) → cleavage of PKCδ (6 hours) → DNA fragmentation and apoptosis (6–24 hours). In contrast, PEP005 activates multiple signaling pathways in these cells, including PKCδ, PKCα, PKCϵ, NF-κB1, ERK, JNK, and Akt. Furthermore, we extended the investigation of AD 198 to TRAF3-sufficient malignant B cells, and found that AD198 also exhibits anti-tumor activity and potently suppresses c-Myc expression in TRAF3-sufficient mouse and human B lymphoma cell lines. Taken together, our findings suggest that AD 198 has therapeutic potential for the treatment of NHL and MM involving TRAF3 inactivation or c-Myc up-regulation.

## Abbreviations

AD 198: N-Benzyladriamycin-14-valerate; PEP005: Ingenol-3-angelate; TRAF3: Tumor necrosis factor receptor (TNF-R)-associated factor 3; B-TRAF3-/-: B cell-specific TRAF3-deficient; NHL: Non-Hodgkin lymphoma; MM: Multiple myeloma; MZL: Splenic marginal zone lymphoma; B-CLL: B cell chronic lymphocytic leukemia; MCL: Mantle cell lymphoma; WM: Waldenström’s macroglobulinemia; LMP1: Latent membrane protein 1; BAFF: B cell activating factor; PKCδ: Protein kinase C δ; NF-κB: Nuclear factor κ light chain enhancer of activated B cells; SHM: Somatic hypermutation; ERK: Extracellular signal-regulated kinase; JNK: c-Jun N-terminal kinase; MTT: 3-(4,5-dimethylthiazol-2-yl)-2,5-diphenyltetrazolium bromide; PI: Propidium iodide; FACS: Fluorescence-activated cell sorting; PCR: Polymerase chain reaction.

## Competing interests

The authors declare that they have no potential conflicts of interest.

## Authors’ contributions

SE and CM designed and performed experiments, analyzed data, and revised the manuscript; YL and SG carried out experiments and analyzed data; LC analyzed data, provided experimental reagents and revised the manuscript; PX supervised and designed this study, analyzed data, and wrote the manuscript. All authors read and approved this manuscript.

## Pre-publication history

The pre-publication history for this paper can be accessed here:

http://www.biomedcentral.com/1471-2407/13/481/prepub

## Supplementary Material

Additional file 1: Figure S1 PEP005 exhibited differential effects on TRAF3^-/-^ mouse B lymphoma and human MM cells. Total viable cell numbers were determined by MTT assay at 24 h after PEP005 treatment. (A and B) Effects on primary splenic B lymphoma cells harvested from diseased B-TRAF3^-/-^ mice. Similar results were also obtained with primary B lymphoma cells purified from ascites, cervical and mesenteric LNs of several individual B-TRAF3^-/-^ mice with tumors. (C) Effects on TRAF3^-/-^ mouse B lymphoma cell lines. (D) Effects on human MM cell lines. The graphs depict the results of three independent experiments with duplicate samples in each experiment (mean ± SEM). **Figure S2.** PEP005 did not induce apoptosis in TRAF3^-/-^ tumor B cells. Cell cycle distribution was determined by PI staining and flow cytometry. TRAF3^-/-^ mouse B lymphoma cell lines (A) or human MM cell lines (B) were cultured in the absence or presence of PEP005 of indicated concentration for 24 h before PI staining. Representative histograms of PI staining are shown, and percentage of apoptotic cells (DNA content < 2n) and proliferating cells (2n < DNA content ≤ 4n) are indicated. Results are representative of three independent experiments. **Figure S3.** Effects of PEP005 on the cytosolic and nuclear levels of PKCδ, NF-κB1 and NF-κB2 subunits, and c-Myc. (A) Dose-dependent effects of PEP005. Mouse or human tumor B cells were cultured with various concentrations of PEP005 for 6 h. (B) Time-dependent effects of PEP005. Mouse or human tumor B cells were cultured in the absence or presence of PEP005 for indicated time periods. Cytosolic and nuclear extracts were prepared as described in the Methods. Proteins were immunoblotted for PKCδ, NF-κB2 (p100 – p52), RelB, NF-κB1 c-Rel, RelA, c-Myc, followed by HDAC1 and actin. Results are representative of three independent experiments. Similar results were also obtained with other TRAF3^-/-^ cell lines. Click here for file
