# Supplementary figures and images for: N-benzyladriamycin-14-valerate (AD 198) exhibits potent anti-tumor activity on TRAF3-deficient mouse B lymphoma and human multiple myeloma
Source: BMC Cancer. 2013 Oct 16;13:481. doi: 10.1186/1471-2407-13-481 (PMC3853153; doi:10.1186/1471-2407-13-481)

Supplementary Figure 1

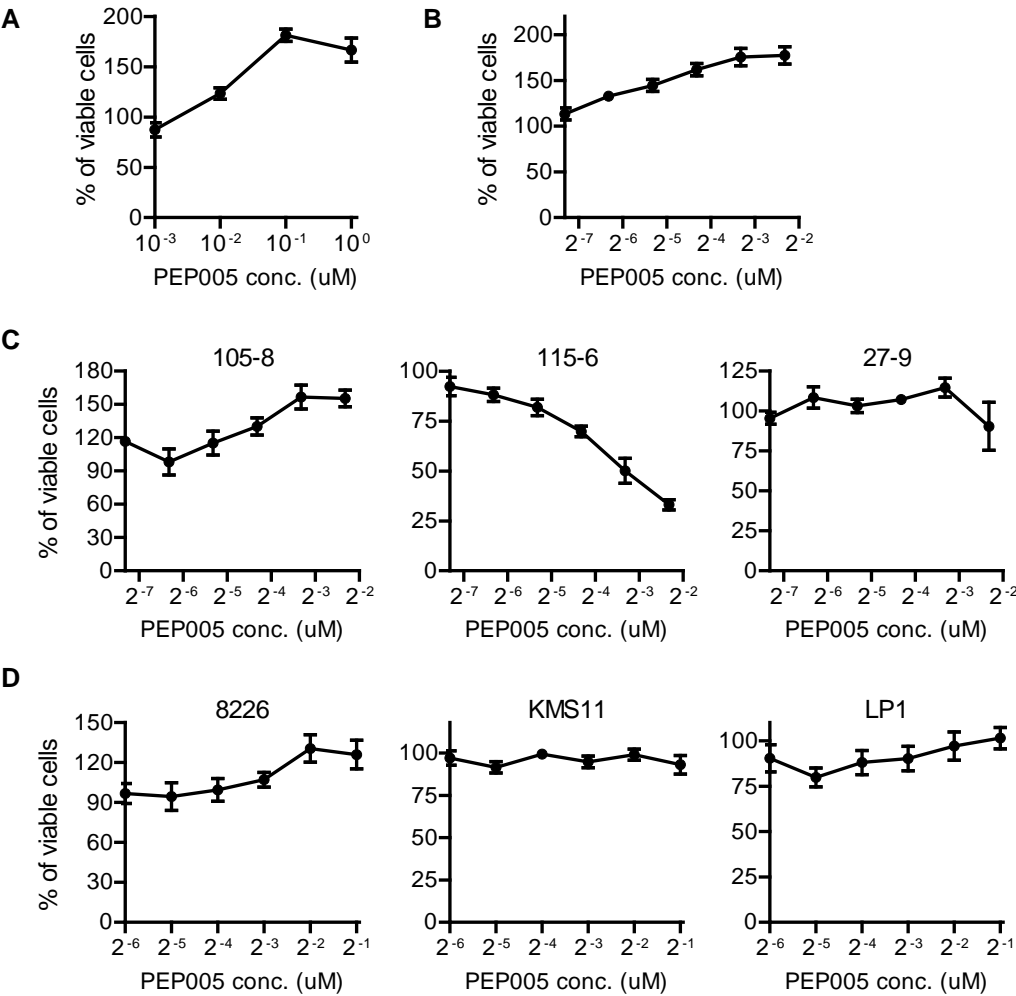

Supplementary Figure 2

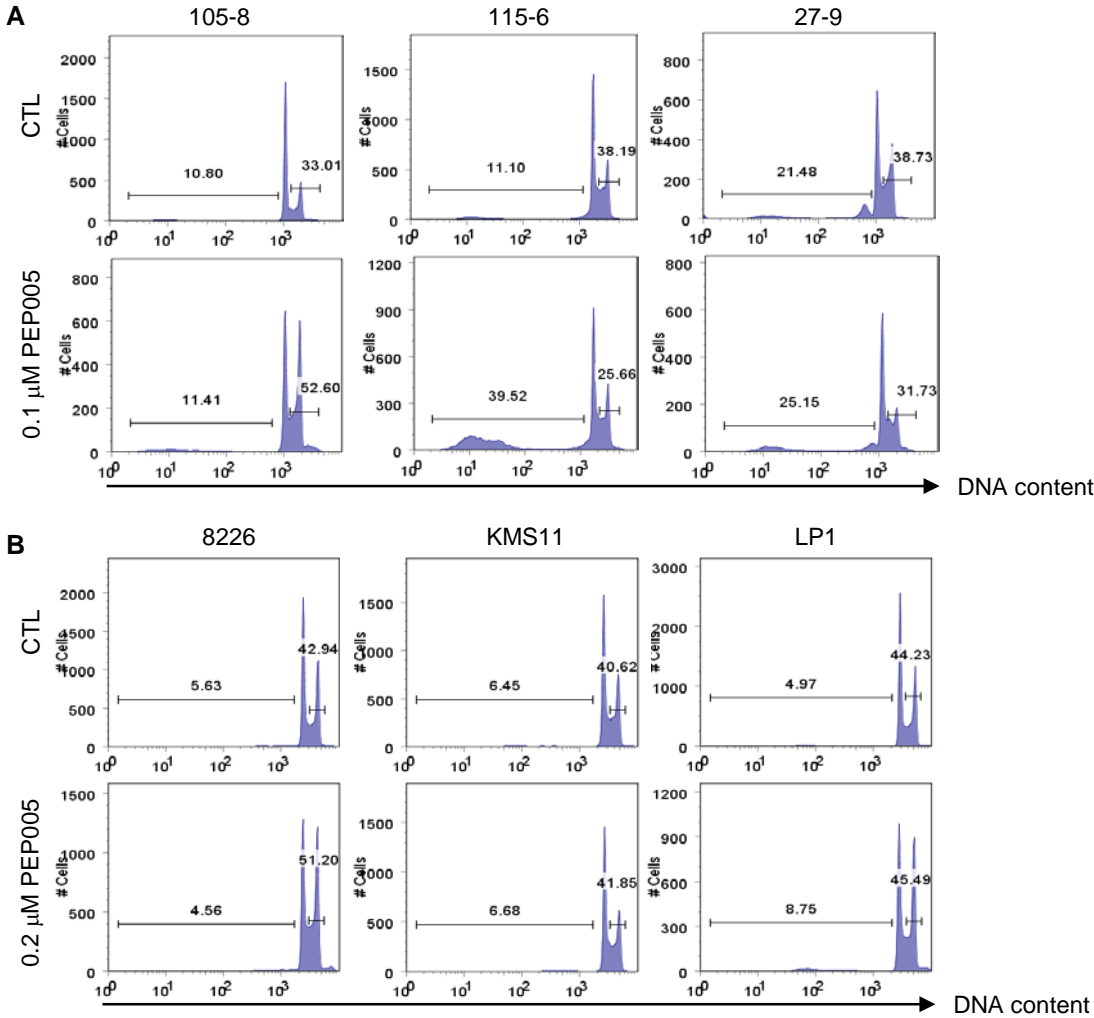

Supplementary Figure 3

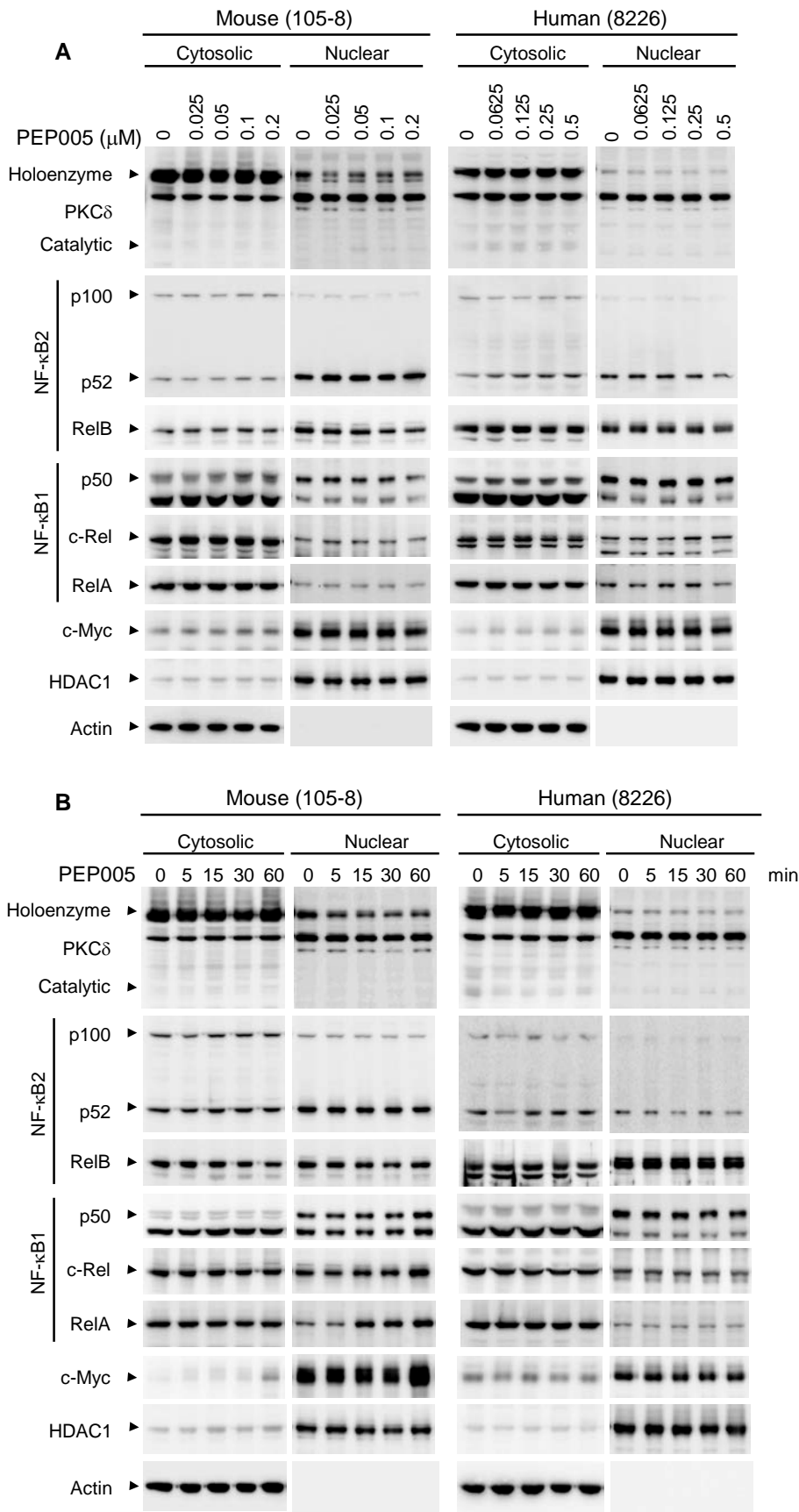

Supplement: Additional file 1: Figure S1 — PEP005 exhibited differential effects on TRAF3-/- mouse B lymphoma and human MM cells. Total viable cell numbers were determined by MTT assay at 24 h after PEP005 treatment. (A and B) Effects on primary splenic B lymphoma cells harvested from diseased B-TRAF3-/- mice. Similar results were also obtained with primary B lymphoma cells purified from ascites, cervical and mesenteric LNs of several individual B-TRAF3-/- mice with tumors. (C) Effects on TRAF3-/- mouse B lymphoma cell lines. (D) Effects on human MM cell lines. The graphs depict the results of three independent experiments with duplicate samples in each experiment (mean ± SEM). Figure S2. PEP005 did not induce apoptosis in TRAF3-/- tumor B cells. Cell cycle distribution was determined by PI staining and flow cytometry. TRAF3-/- mouse B lymphoma cell lines (A) or human MM cell lines (B) were cultured in the absence or presence of PEP005 of indicated concentration for 24 h before PI staining. Representative histograms of PI staining are shown, and percentage of apoptotic cells (DNA content < 2n) and proliferating cells (2n < DNA content ≤ 4n) are indicated. Results are representative of three independent experiments. Figure S3. Effects of PEP005 on the cytosolic and nuclear levels of PKCδ, NF-κB1 and NF-κB2 subunits, and c-Myc. (A) Dose-dependent effects of PEP005. Mouse or human tumor B cells were cultured with various concentrations of PEP005 for 6 h. (B) Time-dependent effects of PEP005. Mouse or human tumor B cells were cultured in the absence or presence of PEP005 for indicated time periods. Cytosolic and nuclear extracts were prepared as described in the Methods. Proteins were immunoblotted for PKCδ, NF-κB2 (p100 – p52), RelB, NF-κB1 c-Rel, RelA, c-Myc, followed by HDAC1 and actin. Results are representative of three independent experiments. Similar results were also obtained with other TRAF3-/- cell lines. [file 1471-2407-13-481-S1.pdf]
